# tLivin Displays Flexibility by Promoting Alternative Cell Death Mechanisms

**DOI:** 10.1371/journal.pone.0101075

**Published:** 2014-06-24

**Authors:** Tamar Shiloach, Christian Berens, Christina Danke, Ortal Waiskopf, Riki Perlman, Dina Ben-Yehuda

**Affiliations:** 1 Division of Hematology, Hadassah-Hebrew University Medical Center, Jerusalem, Israel; 2 Department of Biology/Microbiology, Friedrich-Alexander-Universitaet Erlangen-Nuernberg, Erlangen, Germany; Université de Sherbrooke, Canada

## Abstract

Livin is a member of the Inhibitor of Apoptosis (IAP) protein family that inhibits apoptosis triggered by a variety of stimuli. We previously demonstrated that while Livin inhibits caspase activity, caspases can cleave Livin to produce a truncated protein, tLivin and that this newly formed tLivin paradoxically induces cell death. However to date, the mechanism of tLivin-induced cell death is not fully understood. In this study, we set out to characterize the form of cell death mediated by tLivin. Here we demonstrate that, unlike most death-promoting proteins, tLivin is a flexible inducer of cell death capable of promoting necrosis or apoptosis in different cell lines. The unusual flexibility of tLivin is displayed by its ability to activate an alternative form of cell death when apoptosis is inhibited. Thus, tLivin can promote more than one form of cell death in the same cell type. Interestingly, in cells where tLivin induces necrosis, deletion of the caspase binding BIR domain results in tLivin-induced apoptosis, suggesting the BIR domain can potentially hamper the ability of tLivin to induce apoptosis. We further elucidate that tLivin activates the JNK pathway and both tLivin-induced apoptosis and necrosis are partially mediated by JNK activity. Acquired resistance to apoptosis, common in many tumors, impinges on the efficiency of conventional anti-cancer agents that function primarily by inducing apoptosis. The ability of tLivin to induce death of apoptosis-compromised cells makes it an attractive candidate for targeted cancer therapy.

## Introduction

Programmed cell death plays an important role in development, tissue homeostasis and disease [1]. While apoptosis is a well-characterized form of programmed cell death, necrosis is traditionally perceived as an uncontrolled process. However, growing evidence indicates that necrosis can also constitute a form of programmed cell death activated by specific cues and promoted by cellular signaling pathways [2]–[6]. Acquired resistance to apoptosis is one of the hallmarks of cancer. Traditional cancer treatments such as chemotherapy and γ-irradiation exert their effect by inducing apoptosis; therefore, defects in apoptotic pathways can render cancer cells resistant to therapy. For this reason alternative cell death programs, such as necrosis and autophagy, are studied and their pathways being evaluated as targets for novel therapeutics with important consequences for treatment of apoptosis-resistant tumors [7].

Resistance to apoptosis is often achieved in cancer cells by overexpression of endogenous members of the inhibitors of apoptosis (IAP) protein family [8]. IAPs suppress apoptosis by directly binding and inhibiting both initiator and effector caspases. To date, eight human IAPs have been identified: c-IAP1, c-IAP2, NAIP, Survivin, XIAP, Bruce, ILP-2 and Livin (ML-IAP) [9]. IAP family members contain one to three repeats of the highly conserved BIR (baculovirus IAP repeat) domain at their N-terminus, which mediates their interaction with caspases. Some IAPs contain a RING (really interesting new gene) domain at their C-terminus [10].

We and others identified Livin that contains single BIR and RING domains, as a member of the IAP family [11]–[14]. Livin encodes two splice variants (Livin α and β) with different tissue distribution and different anti-apoptotic properties. The two variants differ by only 18 amino acids located between the BIR and the RING domains, which are present in the Livinα but not in the Livinβ isoform [11]. Livin is a unique IAP that plays a dual role in cell death regulation: Livin blocks apoptosis through its ability to bind and inhibit caspases 3, 7 and 9. Caspases 3 and 7, in turn, cleave Livin during apoptosis at position Asp52 to produce a truncated Livin protein (tLivin). Even though the tLivin protein still harbors the anti-apoptotic BIR domain, it has now acquired a paradoxical death-promoting activity [15]. Thus, Livin is not merely an apoptosis inhibitor but rather a regulator of cell death. We previously demonstrated the dual role of Livin in tumorigenesis in an animal model [16]. Expression of the Livinα isoform promoted tumor growth, while Livinβ inhibited tumor development. This correlated with the cleavage of Livinβ and the subsequent accumulation of the death-promoting tLivinβ in the tumor cells. Indeed, when the death-promoting effect of Livinβ was eliminated by point mutations, the resulting anti-apoptotic Livinβ mutants contributed to tumor progression. Livinα was not cleaved in the tumor cells and thus possessed only anti-apoptotic activity which sustained tumor growth [16].

Livin is upregulated in several malignancies such as gastric carcinoma [17], neuroblastoma [18] and renal cell carcinoma [19]. However, Livin is best linked to melanoma, with high protein levels detected in primary melanoma and in numerous melanoma cell lines, compared with marginal to no Livin protein detectable in melanocytes or naevi [11], [14], [15], [20]. In previous studies, we demonstrated that Livin protein levels were associated with resistance to chemotherapy in primary melanoma cultures [15]. Furthermore, the level of Livin protein was associated with overall survival of melanoma patients. High level of Livin was associated with worse prognosis while, surprisingly, low to intermediate levels of Livin, rather than the complete absence of the protein, was associated with the best prognosis [21]. This favorable prognosis is probably attributed to the death promoting activity of the tLivin protein. Cleavage of Livin and accumulation of tLivin is permissible when Livin is present at low to intermediate levels. Livin is also upregulated in many lung cancer cell lines and primary lung cancer tissues [22], [23] and has been suggested to play a role in the apoptosis resistance of non-small cell lung cancer (NSCLC) cells [24].

c-JUN N-terminal kinase (JNK) is a stress-activated mitogen-activated protein kinase (MAPK). JNK is activated through phosphorylation by the upstream MAPKK proteins MKK4 and MKK7. Following its activation, JNK can phosphorylate a number of transcription factors, such as c-JUN, JunD, ATF2, c-Myc and p53 [25], as well as other cytoplasmic proteins including members of the Bcl-2 family [26], [27]. Activation of the JNK pathway may result in cell survival or cell death in different settings. It is suggested that an early and transient activation of JNK mediates cell survival, while a late and persistent activation of JNK mediates cell death [28], [29]. The JNK pathway promotes both apoptosis and necrosis [30]–[34].

In this study, we examined the mechanism of cell death induced by tLivinβ. We show that tLivin induces distinct forms of cell death in cells of different origin: a caspase-independent necrotic cell death of 293T cells and apoptosis of melanoma and lung cancer cell lines. Additionally, we show that tLivinΔBIR, a tLivin mutant lacking its caspase binding domain, induces apoptosis of 293T cells, unlike wild type tLivin. Interestingly, both tLivin and tLivinΔBIR can activate an alternative form of cell death when apoptosis is inhibited. We also demonstrate that tLivin induces the activation of the JNK pathway and that both tLivin-induced necrosis and apoptosis are partially mediated by JNK activity.

## Materials and Methods

### Cells

293T human embryonic kidney cells (ATCC) and LB33 MelA1 human melanoma cells (a kind gift from Prof. PG. Coulie, Université Catholique de Louvain, Brussels, Belgium [35]) were grown in DMEM. A549 human lung non-small carcinoma cells, (provided by Prof. Shosh Ravid, The Hebrew University of Jerusalem, Jerusalem, Israel [36]) were grown in RPMI 1640. Media were supplemented with 10% fetal calf serum, 100 U/ml penicillin, 100 µg/ml streptomycin, 1 mM L-glutamine and 1 mM sodium pyruvate. Cells were grown at 37°C in a humidified atmosphere containing 5% CO_2_.

### Reagents

Sodium azide, anisomycin and doxycycline (dox) were purchased from Sigma (St Louis, MO, USA). zVAD-fmk, SP600125 and staurosporine were purchased from Enzo Life Sciences (Farmingdale, NY, USA). Etoposide was purchased from EBEWE Pharma (Kundl, Austria) and cisplatin was purchased from TEVA ABIC (Netanya, Israel).

### Plasmids and transfections

The pIRES2-EGFP plasmid encoding for tLivin was described elsewhere [15]. The tLivinΔBIR mutant lacks amino acids 86–158 encompassing the BIR domain. tBID was sub-cloned from pcDNA3 (a kind gift from Prof. A Gross, Weizmann Institute of Science, Rehovot, Israel) into pIRES2-EGFP. pCMV-FLAG-JIP1 was a kind gift from Prof. RJ Davis (University of Massachusetts Medical School, Worcester, MA, USA).

293T cells were transfected by the calcium phosphate method. A549 cells and MelA1 cells were transfected with JetPrime (Polyplus Transfection, Illkirch, France) and FugeneHD (Roche Diagnostics GmbH, Mannheim, Germany), respectively, according to the manufacturers’ instructions.

### Establishment of a Tet-On inducible system for the expression of tLivin

The pWHE644 plasmid (described elsewhere [37], [38]), encoding the reverse transactivator rtTA2^s^-M2, the transsilencer tTS^D^-PP and a resistance marker for puromycin, as well as the pWHE655-TREtight plasmid, encoding an insulated inducible expression cassette and a resistance marker for neomycin (described elsewhere [39]), were used to establish an inducible expression system in cultured cells. tLivin was sub-cloned into pWHE655.

A549 cells were transfected with the pWHE644 plasmid and puromycin (Invivogen, San Diego, CA, USA) was added 48 h post-transfection (0.4 µg/ml). Cells from each resistant clone were transiently transfected with pWHE655-GFP and screened for dox-induced expression of GFP. Positive clones were transfected with pWHE655-tLivin and selected with 700 µg/ml G418 (Invitrogen, Carlsbad, CA, USA). G418-resistant clones were examined for dox-induced expression of tLivin.

MelA1 cells were co-transfected with pWHE644 and pWHE655-tLivin. Both 0.25 µg/ml of puromycin and 125 µg/ml of G418 were added to the culture 48 h post-transfection. Resistant MelA1 clones were isolated, grown individually and screened for dox-induced expression of tLivin.

### Cell death analysis

Measurements of cell death were performed using two different methods:

1. To quantify the percent of cell death, cells were harvested, centrifuged at 800 g for 7 min and the pellet was resuspended in PBS with 10 µg/ml of PI (propidium iodide [Sigma]). PI-positive stained cells were quantified by flow cytometry (FL-3 channel). In transiently transfected 293T cells, GFP expression was analyzed first (FL-1) and only GFP-positive cells were analyzed for PI staining.
2. For the cell cycle assay, cells were harvested, washed with PBS and fixed in 70% ethanol at −20°C for at least 2 h. Cells were then centrifuged at 800 g for 5 min, and the pellet was resuspended in PBS and incubated on ice for 30 min. Following additional centrifugation, the pellet was resuspended in PBS containing 50 µg/ml RNase A (Sigma) and 5 µg/ml PI and incubated at 37°C for 30 min. The stained cells were analyzed for PI fluorescence (FL2-A channel), and the percent of cells in the SubG1 fraction was determined.

Flow cytometry was performed with a FACSCalibur (BD Biosciences, San Jose, CA, USA) and data was analyzed with CellQuest Pro software (BD Biosciences). Results are expressed as the mean ± standard deviation (SD) from three independent experiments.

### Western blot analysis and antibodies

Cells were lysed in RIPA buffer (50 mM Tris-HCl pH 7.5, 150 mM NaCl, 1% Nonidet P-40 (NP-40), 0.1% SDS, 0.5% (w/v) sodium deoxycholate and protease inhibitors: 1 mM PMSF and complete inhibitor cocktail [Roche]) on ice for 30 min. Lysates were centrifuged at 20,000 g for 15 min and the supernatant was collected. Protein content was measured by the DC protein assay (Bio-Rad, Hercules, CA, USA) according to the manufacturer’s instructions. Equal amounts of protein from each sample were resolved on 12% Bis-Tris pre-cast gels (Invitrogen) and transferred to a PVDF membrane (Millipore, Bellerica, MA, USA). Membranes were blocked with 5% low-fat milk in PBS supplemented with 0.1% Tween 20 (PBST) and then incubated with various primary antibodies followed by a secondary horseradish peroxidase-conjugated antibody. Membranes were washed after the primary and secondary antibodies three times in PBST.

The antibody for Livin was purchased from Imgenex (San Diego, CA, USA). Antibodies for caspase 3, caspase 9, P-JNK, JNK, P-ATF2, ATF2, c-JUN, P-p38, p38 and β-Actin were purchased from Cell Signaling Technology (Beverly, MA, USA). Antibodies for P-c-JUN, JIP-1 and GAPDH were purchased from Santa Cruz Biotechnology (Santa Cruz, CA, USA). The antibody for PARP was purchased from Enzo Life Sciences.

### Transmission electron microscope

293T cells were fixed for 1 h in Karnovsky’s fixative (3% paraformaldehyde, 2% glutaraldehyde, 5 mM CaCl_2_ in 0.1 M cacodylate buffer [pH 7.4] and 0.1 M sucrose). Cells were scraped, pelleted and embedded with agar noble to a final concentration of 1.7% and postfixed with 1% OsO_4_, 0.5% potassium dichromate and 0.5% potassium hexacyanoferrate in 0.1 M cacodylate buffer. The pellet was stained en bloc with 2% aqueous uranyl acetate followed by ethanol dehydration and embedded in EMbed (EMS). Sections (75 nm) were cut, stained with 2% uranyl acetate in 50% ethanol and lead citrate, and examined using a FEI CM12 EINDHOVEN transmission electron microscope at an accelerating voltage of 120 kV. Digital images were obtained with MegaView3 CCD camera (SIS GMBH).

## Results

### tLivin induces distinct forms of cell death in different types of cells

**Figure 1:**
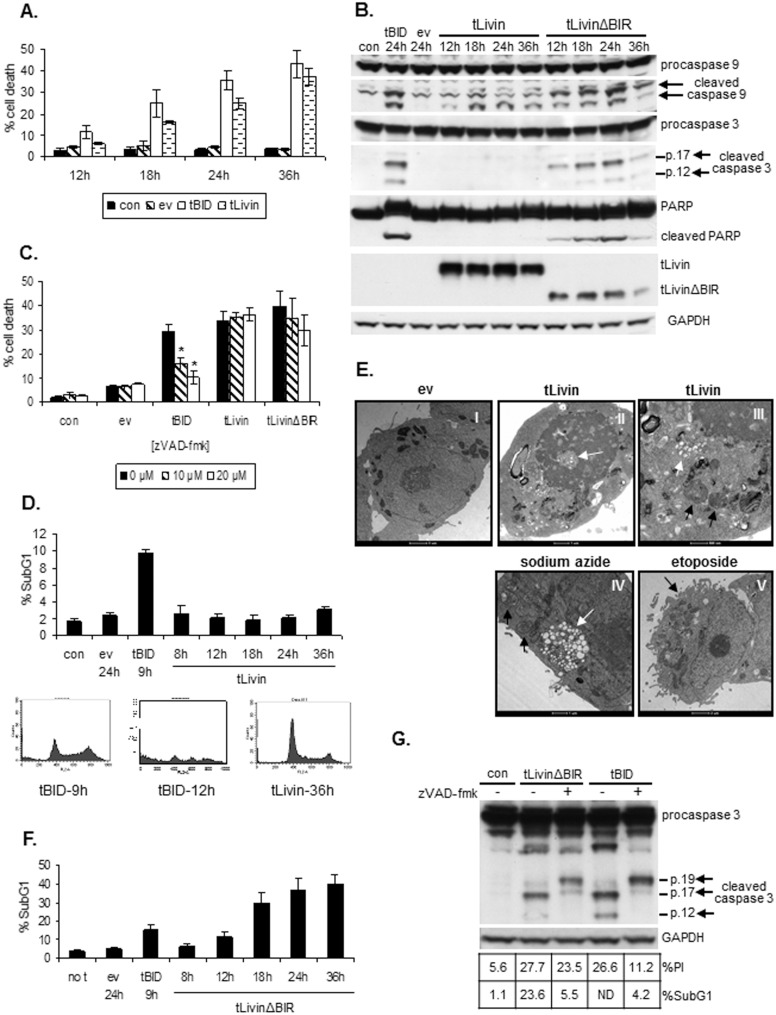
tLivin induces caspase-independent necrotic cell death of 293T cells. 293T cells were transfected with an empty vector (ev) or with vectors expressing either tBid, tLivin or tLivinΔBIR and harvested at the indicated time points post-transfection. Con, untransfected cells; (**A**) Cells were stained with propidium iodide (PI) and the percent of cell death was measured by flow cytometry. (**B**) Cells were analyzed by western blot for cleavage of caspases and PARP. (**C**) zVAD-fmk was added 1 h before transfection. Cells were harvested 24 h post-transfection and stained with PI. The percent of cell death was measured by flow cytometry. *p<0.05. (**D**) Cells were analyzed for DNA content by flow cytometry (top). Representative cell cycle histograms of tBID- and tLivin-transfected cells (bottom). (**E**) Cells were examined by transmission electron microscopy 18 h post-transfection with empty vector (I) or a tLivin-expressing vector (II, III). Cells were incubated with 10 mg/ml sodium azide for 4 h (IV) or 30 µg/ml etoposide for 24 h (V). Features of necrosis: cytoplasmic vacuolation (white arrow) (II, III, IV) and rounded mitochondria with disrupted internal structures (black arrows) (III, IV) are evident in cells transfected with tLivin and in cells treated with sodium azide. Apoptotic cells (treated with etoposide) exhibited blebbing of the plasma membrane (black arrow) (V). (**F**) Cells were analyzed for DNA content by flow cytometry. (**G**) zVAD-fmk at 20 µM was added 1 h before transfection and cells were harvested 24 h post-transfection. Each sample was divided into three aliquots and analyzed for cleavage of caspase 3 by western blot and for the percent of PI positive cells and DNA content by flow cytometry. ND, Not determined.

To characterize the type of cell death induced by tLivin in 293T cells, tLivin was ectopically expressed in these cells, leading to a time-dependent increase in cell death which reached 37.3% (±3.7) death at 36 hours (h) post-transfection. The kinetics of tBID-induced cell death was also determined (as a positive control for apoptosis) and showed similar percent of cell death at 36 h (Figure 1A). We first examined two hallmarks of apoptosis, activation of caspases and fragmentation of DNA. Ectopic expression of tLivin resulted in activation of caspase 9, but not of the downstream caspase 3, the major executioner of apoptosis. Additionally, the caspase substrate, PARP, was not cleaved in cells expressing tLivin. In contrast, tBID overexpression led to robust activation of both caspases as well as to cleavage of PARP (Figure 1B). Furthermore, the pan-caspase inhibitor, zVAD-fmk, did not block tLivin-induced cell death while it significantly inhibited apoptotic cell death induced by tBID in a dose-dependent manner (p<0.05) (Figure 1C). Approximately 10% of the cells transfected with tBID were sub-diploid as early as 9 h post-transfection (Figure 1D top). A ‘collapse’ of the cell cycle did not allow quantification of the DNA content at later time points (Figure 1D, bottom histograms). In contrast, cells transfected with tLivin retained a classical cell cycle histogram throughout the experiment and were not sub-diploid even at 36 h post-transfection (Figure 1D). Altogether, these results indicate that tLivin does not induce apoptosis of 293T cells, but rather a distinct, caspase-independent form of cell death.

To determine the type of cell death induced by tLivin in 293T cells, we examined the morphology of cells expressing tLivin by transmission electron microscopy (Figure 1E). Treatment of cells with sodium azide or etoposide served as positive controls for necrosis and apoptosis, respectively. Cells transfected with tLivin exhibited marked features of necrosis, mainly vacuolation of the cytoplasm and rounded mitochondria with abnormal cristae structure (Figure 1E, II, III). The same features were dominant in cells undergoing necrosis induced by sodium azide (Figure 1E, IV). Following exposure to etoposide, apoptotic cells exhibited significant blebbing of the plasma membrane, an important characteristic of apoptosis (Figure 1E, V); in contrast, there was no blebbing of the plasma membrane of cells expressing tLivin (Figure 1E, II). Swelling of the mitochondria, a characteristic of necrosis, was observed in tLivin-induced cell death, but predictably not seen in cells undergoing apoptosis (Figure 1E, V). These results indicate that indeed tLivin does not induce apoptosis of 293T cells rather; tLivin induces necrosis of 293T cells.

We further examined whether necrosis of 293T cells induced by tLivin is in fact necroptosis, a form of programmed necrosis mediated by RIP1 kinase and MLKL [7]. The addition of specific RIP1 kinase and MLKL inhibitors, necrostatin-1 and necrosulfonamide, respectively, did not inhibit tLivin-induced death of 293T cells (data not shown), suggesting this form of cell death is not necroptosis.

Cleavage of Livin by caspases produces a tLivin protein that retains its BIR domain known to mediate caspase binding and inhibition [15]. We questioned whether the inhibitory effect of the BIR domain on caspase activity prevented tLivin from inducing apoptosis. We generated a tLivin mutant lacking the BIR domain and explored the form of cell death induced by this tLivinΔBIR mutant in 293T cells. Interestingly, tLivinΔBIR induced robust activation of both caspase 9 and 3, equivalent to the level of caspase activation induced by tBID, as well as cleavage of PARP (Figure 1B). The percent of cells in the sub-diploid fraction gradually increased following transfection of tLivinΔBIR and reached 40.2% (±4.5%) at 36 h post-transfection (Figure 1 F). These results indicate that, unlike wild type tLivin, which induces necrosis, the tLivinΔBIR mutant induces apoptosis of 293 T cells.

**Figure 2:**
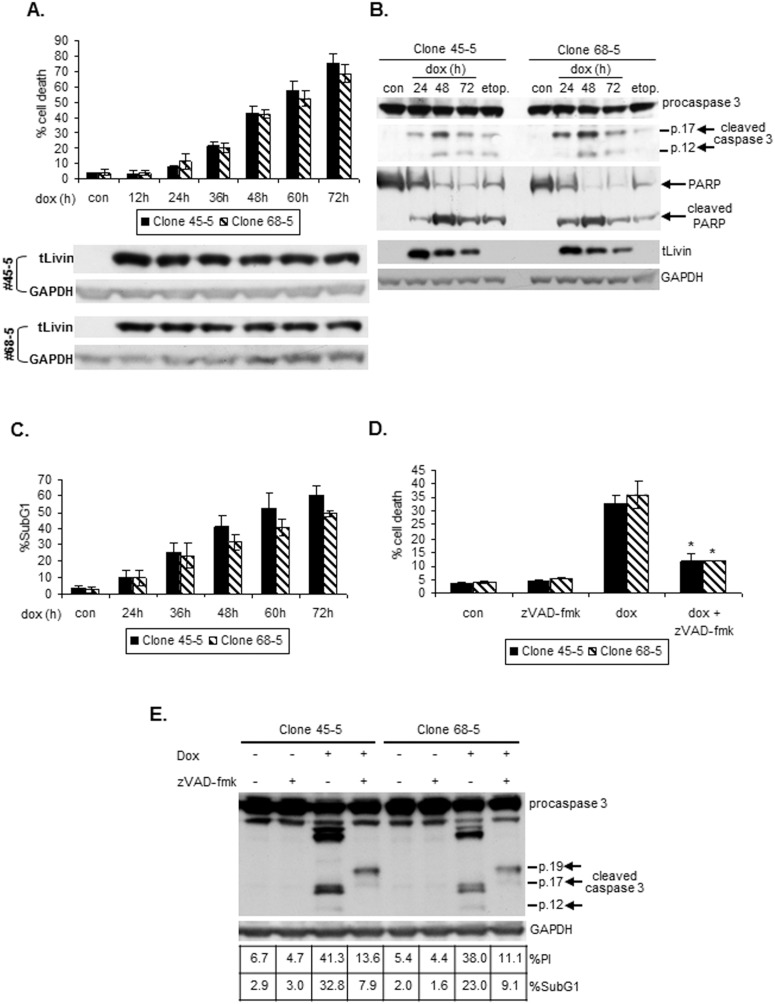
tLivin induces apoptosis of A549 cells. Cells of A549 clones were treated with 2.5 µg/ml dox and harvested at the indicated time points. Con, untreated cells; etop, cells treated with 30 µg/ml etoposide for 48 h. (**A**) Cells were stained with PI and the percent of cell death was measured by flow cytometry (top). Cells were analyzed for tLivin and GAPDH protein levels by western blot (bottom). (**B**) Cleavage of caspase 3 and PARP was analyzed by western blot. (**C**) DNA content was analyzed by flow cytometry. (**D**) Cells were treated with 50 µM zVAD-fmk 1 h prior to addition of dox, harvested after 48 h, stained with PI and the percent of cell death was measured by flow cytometry. *p<0.05. (**E**) Cells were treated with 50 µM zVAD-fmk 1 h prior to addition of dox and harvested after 48 h. Each sample was divided into three aliquots and analyzed for cleavage of caspase 3 by western blot and for the percent of PI positive cells and DNA content by flow cytometry.

**Figure 3:**
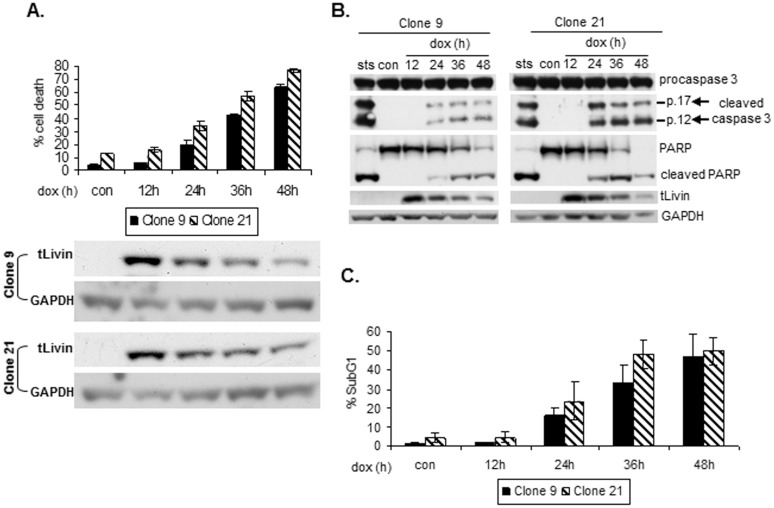
tLivin induces apoptosis of MelA1 cells. Cells of MelA1 clones were treated with 2.5 µg/ml dox and harvested at the indicated time points. Con, untreated cells; sts, cells treated with 0.5 µM staurosporine for 10 h. (**A**) Cells were stained with PI and the percent of cell death was measured by flow cytometry (top). Cells were analyzed for tLivin and GAPDH protein levels by western blot (bottom). (**B**) Cleavage of caspase 3 and PARP was analyzed by western blot. (**C**) DNA content was analyzed by flow cytometry.

Livin protein is aberrantly expressed in many malignancies [8]. We therefore characterized tLivin-induced cell death in cell lines representing tumors in which Livin overexpression has been documented, lung cancer and melanoma. tLivin was expressed via a stringently-controlled Tet-On inducible system in A549, a lung cancer cell line, and in MelA1, a melanoma cell line (see materials and methods). Both cell lines lack endogenous expression of Livin protein permitting the expression of tLivin and analysis of its activity to be defined. Two clones were chosen from each cell line in which expression of tLivin was induced by the administration of doxycycline (dox) (see materials and methods). tLivin protein expression was induced in both A549 and MelA1 cells to maximal levels at 12 h post dox administration (Figures 2A and 3A). Treatment of cells with dox and the subsequent accumulation of the tLivin protein resulted in a time-dependent increase in the percent of cell death (Figures 2A and 3A). In contrast to 293T cells, caspase 3 was robustly activated and cleavage of PARP was observed in both A549 and MelA1 cells after induction of tLivin expression (Figures 2B and 3B). Additionally, DNA fragmentation measured by the percent of cells in the sub-diploid fraction, increased with time following induction of tLivin expression (Figures 2C and 3C) and correlated well with the kinetics of cell death (Figures 2A and 3A). Taken together, these results indicate that despite the presence of its BIR domain, tLivin induces apoptosis of A549 and MelA1 cells, unlike the necrotic cell death it induces in 293T cells.

### tLivin and tLivinΔBIR activate an alternative form of cell death when apoptosis is inhibited

**Figure 4:**
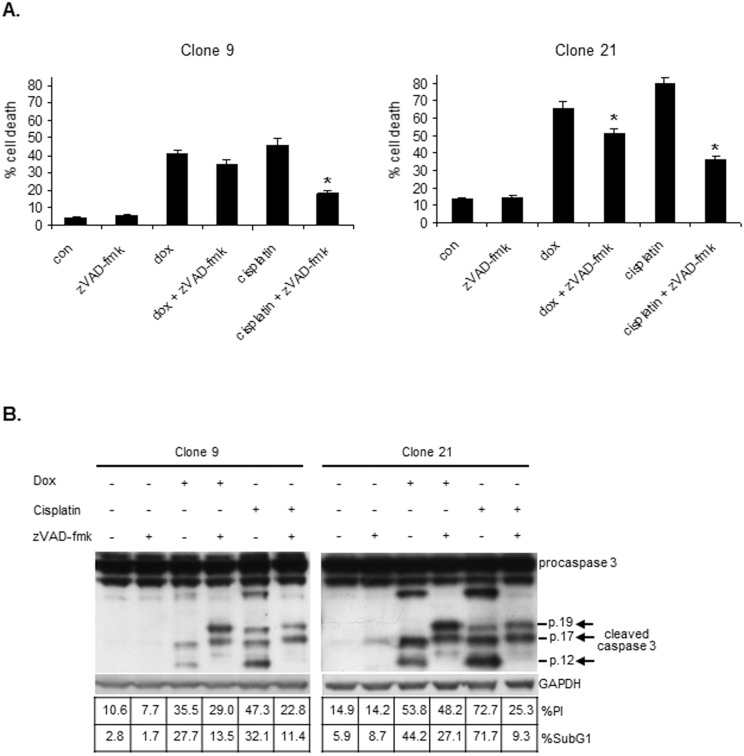
tLivin activates an alternative form of cell death when apoptosis is inhibited. Cells of MelA1 clones were treated with 75 µM zVAD-fmk 1 h prior to addition of 2.5 µg/ml dox or 30 µM cisplatin and harvested after 36 h along with untreated cells (con). (**A**) Cells were stained with PI and the percent of cell death was measured by flow cytometry. *p<0.05. (**B**) Cells from each sample were divided into three aliquots and analyzed for cleavage of caspase 3 by western blot and for the percent of PI positive cells and DNA content by flow cytometry.

We showed above that tLivin induces apoptosis of A549 and MelA1 cells. Indeed, the pan-caspase inhibitor zVAD-fmk significantly (p<0.05) inhibited tLivin-induced cell death of A549 cells: 64.1% (±6.9%) and 67.0% (±4.8%) inhibition of cell death in clones 45-5 and 68-5, respectively (Figure 2D). Unexpectedly, when we blocked apoptosis induced by tLivin in MelA1 cells by zVAD-fmk, we observed that inhibition of caspase activity only partially repressed tLivin-induced cell death of MelA1 cells: 14.7% (±3.6%) and 21.6% (±0.5%) inhibition in clone 9 and clone 21, respectively (Figure 4A). In contrast, apoptosis induced by cisplatin, a chemotherapeutic drug, was dramatically inhibited by the addition of zVAD-fmk: 59.4% (±7.2%) and 54.9% (±5.2%) inhibition in clone 9 and clone 21, respectively (Figure 4A). In 293T cells, cell death induced by tLivinΔBIR was only partially and non-significantly inhibited by zVAD-fmk, resulting in 24.9% (±3.8%) decrease in percent cell death with the addition of 20 µM of inhibitor (Figure 1C). However, cell death induced by tBID was markedly decreased by the addition of zVAD-fmk, resulting in significant 45.3% (±7.6%) and 65.4% (±8%) inhibition of cell death at concentrations of 10 µM and 20 µM, respectively (Figure 1C).

To confirm that zVAD-fmk effectively inhibited apoptosis, we examined the correlation between cell death (the percent of PI-positive cells) and apoptosis, using two apoptotic markers: 1) DNA fragmentation (the percent of cells in the sub-diploid fraction) and 2) cleavage of caspase 3. In A549 cells expressing tLivin, addition of zVAD-fmk inhibited both apoptosis and cell death (Figure 2E). In contrast, in MelA1 cells, zVAD-fmk only slightly inhibited tLivin-induced cell death despite an efficient repression of apoptotic markers manifested by incomplete cleavage of caspase 3, as well as a marked (∼40–50%) decrease in the percent of cells in the sub-diploid fraction (Figure 4B). Similarly, in 293T cells expressing tLivinΔBIR, addition of zVAD-fmk led to inhibition of apoptosis including a dramatic decrease (76.7%) in the percent of cells in the sub-diploid fraction, as well as incomplete cleavage of caspase 3, with only a marginal effect on the percent of dead cells (Figure 1G).

These results show that while tLivin and tLivinΔBIR induce apoptosis of MelA1 and 293T cells, respectively, they can also induce an alternative form of cell death when the apoptotic pathway is blocked. Thus, tLivin and tLivinΔBIR can induce more than one type of cell death in the same cell line.

### tLivin activates the JNK pathway

The activation of the JNK MAPK pathway is linked to cellular survival as well as to the promotion of cell death [40]. Overexpression of full length Livin has been shown to inhibit apoptosis through activation of JNK [41], [42]. Given the dual role of JNK in life and death of cells, and the ability of full length Livin to activate JNK, we explored whether JNK can also be activated by tLivin.

**Figure 5:**
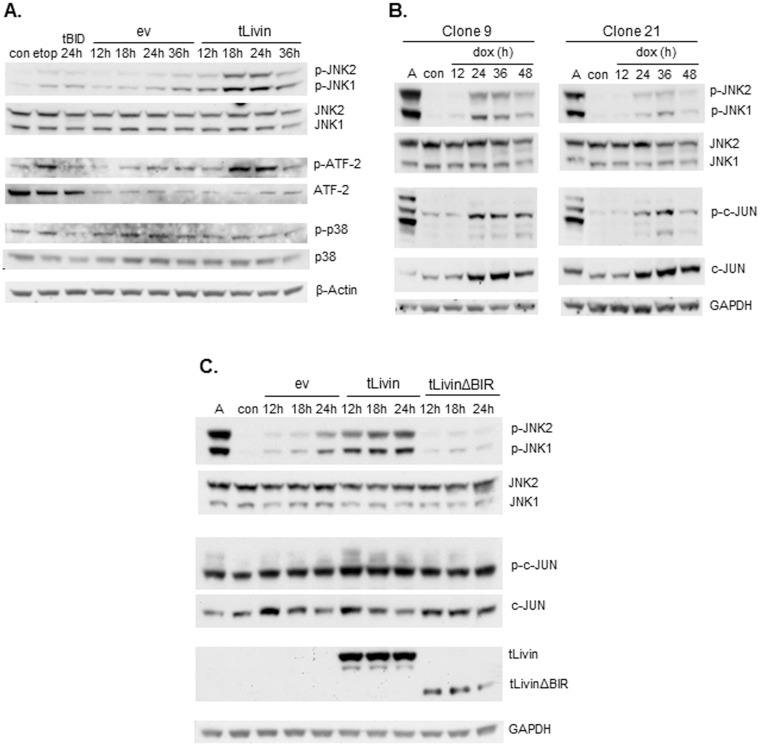
tLivin activates the JNK pathway. (**A**) 293T cells were either transiently transfected with empty vector (ev), tBID- or tLivin-expressing vectors, treated with 30 µg/ml etoposide for 24 h (etop) or left untreated (con). Cells were harvested at the indicated time points post-transfection and the phosphorylation of JNK, ATF-2 and p38MAPK was analyzed by western blot. (**B**) Cells of MelA1 clones were harvested at the indicated time points following administration of 2.5 µg/ml dox or following 30 min treatment with 200 nM anisomycin (A), control cells (con) were left untreated. Phosphorylation of JNK and c-JUN was analyzed by western blot. (**C**) 293T cells were either transiently transfected with empty vector (ev), tLivin- or tLivinΔBIR-expressing vectors, treated with 200 nM anisomycin for 30 min (A) or left untreated (con). Cells were harvested at the indicated time points post-transfection and phosphorylation of JNK and c-JUN was analyzed by western blot.

In 293T cells transiently transfected with tLivin, the levels of phosphorylated JNK1 and JNK2 elevated considerably at 18 h and 24 h post-transfection (Figure 5A), whereas, the total levels of JNK proteins remained constant in all treatments and transfections tested. Phosphorylation of the JNK substrate, ATF-2, correlated with the phosphorylation state of JNK (Figure 5A). These results indicate that tLivin induces the activation of the JNK pathway in 293T cells. To examine the specificity of JNK activation we tested the phosphorylation state of p38, another MAPK known to promote cell death [40], and found that it was not activated by tLivin (Figure 5A). This finding suggests a specific role for JNK in tLivin-induced cell death of 293T cells. The activation of JNK appears to precede the peak time of cell death induction in 293T cells transfected with tLivin as JNK1/2 phosphorylation levels peak at 18 h post-transfection (Figure 5A) when the rate of cell death is only 16.3% (±0.7%) (Figure 1A).

JNK was also activated in MelA1 cells after induction of tLivin. In both clones, JNK phosphorylation was first detected 24 h after dox administration (Figure 5B), in correlation with the onset of cell death (Figure 3A). JNK phosphorylation coincided with the phosphorylation of its substrate, c-JUN and with increased levels of total c-JUN, probably due to c-JUN’s autoregulation of its transcription [43] (Figure 5B). Anisomycin, a potent activator of JNK, which was used as a positive control, is an inhibitor of protein synthesis and therefore, despite strong phosphorylation of c-JUN in cells treated with anisomycin, no increase in total c-JUN protein levels was detected. Interestingly, the tLivinΔBIR mutant was unable to activate JNK (Figure 5C). Although tLivinΔBIR expression was markedly lower than the expression of wild type tLivin (Figure 5C), both proteins induce comparable levels of cell death when transfected into 293T cells (Figure 1C, black bars), suggesting that the BIR domain is required for the activation of JNK by tLivin.

### tLivin-induced apoptosis and necrosis are partially mediated by activation of JNK

**Figure 6:**
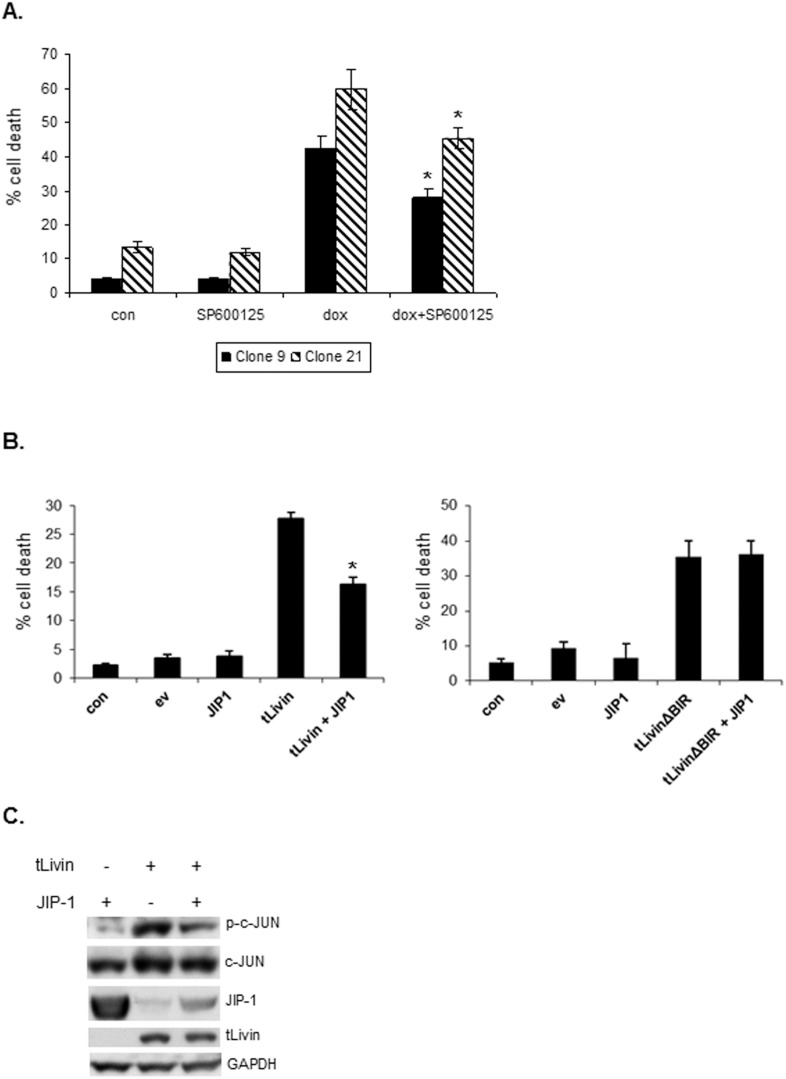
tLivin-induced apoptosis and necrosis are partially mediated by activation of JNK. (**A**) Cells of MelA1 clones were treated with 10 µM SP600125 1 h prior to administration of 2.5 µg/ml dox (where indicated) and harvested after 36 h along with untreated cells (con). Cells were stained with PI and the percent of cell death was measured by flow cytometry. *p<0.05. (**B**) 293T cells were transiently transfected with the indicated plasmids and harvested 24 h post-transfection along with untransfected cells (con). Cells were stained with PI and the percent of cell death was measured by flow cytometry. (**C**) 293T cells were transiently transfected with the indicated plasmids, harvested 24 h post-transfection and analyzed by western blot for the protein levels of p-c-JUN, c-JUN, JIP-1, tLivin and GAPDH.

We next determined whether activation of the JNK pathway is essential for tLivin-induced apoptosis or necrosis. Therefore, MelA1 cells were treated with the JNK specific inhibitor, SP600125. In both MelA1 clones, SP600125 led to a partial, yet statistically significant (p<0.05), inhibition of tLivin-induced cell death: 34.3% (±7.2%) and 23.8% (±2.6%) inhibition of cell death in clones 9 and 21 respectively (Figure 6A). Since we found that SP600125 was toxic to 293T cells, inhibition of the JNK pathway was performed by overexpressing the scaffold protein JIP-1 which regulates the activation of JNK by interacting with components of the JNK signaling pathway [44]. When overexpressed, JIP-1 causes cytoplasmic retention of JNK and prevents the phosphorylation of nuclear JNK substrates [45]. Indeed, co-transfection of tLivin with JIP-1 resulted in inhibition of c-JUN phosphorylation (Figure 6C) and a significant 41.1% (±6.6%) inhibition of cell death induced by tLivin in 293T cells (Figure 6B). Of note, overexpression of JIP-1 still allows the phosphorylation of cytoplasmic substrates of JNK [46]. Interestingly, co-expression of tLivin with JIP-1 led to a marked decrease in JIP-1 protein level compared with cells expressing JIP-1 alone (Figure 6C). The RING domain of tLivin has a putative E3-ubiquitin ligase activity [47] which could mediate the degradation of JIP-1. Since tLivinΔBIR does not induce the activation of JNK (Figure 5C), overexpression of JIP-1 did not inhibit tLivinΔBIR-induced cell death (Figure 6B). These results indicate that both apoptosis of MelA1 cells and necrosis of 293T cells induced by tLivin are partially mediated through the JNK pathway.

## Discussion

Tumorigenesis is a multistep process involving genetic alterations that confer an advantage to transformed cells. Perhaps the most prominent alteration of all is the acquired resistance to apoptosis, which occurs in almost all types of cancer and constitutes an important clinical hurdle in the treatment of cancer. Consequently, the type of cell death induced by an anti-cancer agent may have significant impact on the efficiency of treatment.

In this study, we revealed that tLivin induces distinct forms of cell death in cancer cells of differing origins. In 293T cells, ectopic expression of tLivin induces a caspase-independent necrotic cell death (Figure 1). Interestingly, in a melanoma and a lung cancer cell line tLivin induces apoptosis, rather than necrosis (Figures 2 and 3). A similar phenomenon has been documented in the literature for other cell death stimuli. For example, Bnip3, a member of the Bcl-2 protein family, induces distinct forms of cell death in different types of cell lines: necrosis of 293T and A549 cells [48], [49] and apoptosis of MCF-7 breast cancer cells [50]. Similarly, doxorubicin, a topoisomerase II inhibitor, induces apoptosis of MCF-7 cells and PA-1 cells, human ovarian teratocarcinoma cells; however, it induces necrosis of Jurkat cells, an acute T cell leukemia cell line [51], [52]. Furthermore, doxorubicin also exhibits cardiotoxicity due to induction of both apoptosis and necrosis of cardiomyocytes [53].

To address the role of p53 in the cell death response to tLivin expression, we examined the correlation between the status of p53, a master regulator of apoptosis [54], and the form of cell death induced by tLivin in each cell line. Expression of SV40 large T antigen in 293T cells renders p53 inactive and these cells undergo necrosis following transfection of tLivin. Conversely, both A549 and MelA1 cells express wild type p53, as we determined by sequencing of the hot spot regions of p53 (exons 4–10). Unlike 293T cells, A549 and MelA1 cells undergo apoptosis when induced to express tLivin. Interestingly, the tLivinΔBIR mutant induces apoptosis of 293T cells despite the lack of active p53. Further examination of the mechanism of tLivin-induced cell death in a variety of cancer cells differing in their p53 status will help to fully understand the correlation.

We have also shown that tLivin can induce more than one type of cell death within the same cancer cell line. We found that apoptosis is the default mechanism of cell death induced by tLivin in MelA1 cells. However, tLivin can induce an alternative form of cell death when activation of caspases is inhibited (Figure 4). Tumor cells acquire resistance to apoptosis by various mechanisms that interfere at different levels of apoptotic signaling, such as overexpression of anti-apoptotic genes (Bcl-2, IAPs) [55], [56] and alteration in the signaling pathways of tumor suppressors and oncogenes (p53, Akt) [57], [58]. Inhibition of caspases by zVAD-fmk mimics the various types of acquired resistance to apoptosis, characteristic of cancer cells, and demonstrates the important ability of tLivin to overcome resistance to apoptosis. Like tLivin, several anti-cancer drugs such as imatinib mesylate, staurosporine and cycloheximide were shown to induce necrosis of cancer cells when apoptosis was inhibited [59]–[61].

The interaction of IAPs with caspases has the potential to regulate not only caspase activity but IAP function as well. The anti-apoptotic activity of XIAP, c-IAP1 and Livin is regulated during apoptosis through specific cleavage by effector caspases [15], [62], [63]. Caspase-mediated cleavage of Livin and c-IAP1 generates death-promoting factors out of anti-apoptotic proteins. The pro-apoptotic truncated c-IAP1 lacks the three BIR domains [63] whereas Livin is cleaved by caspases at Asp52 resulting in a tLivin protein that retains the BIR domain [15]. The BIR domain binds and inhibits caspases and indeed tLivin was shown to interact with caspase 3 [64]. The uniqueness of tLivin is that it induces apoptosis of A549 and MelA1 cells despite the presence of the BIR domain. In 293T cells, tLivin induces a non-apoptotic cell death implying that in some cellular settings the presence of the BIR domain may impinge on the ability of tLivin to induce apoptosis. Indeed, we show that the tLivinΔBIR mutant, lacking the caspase binding domain, induces apoptosis of 293T cells (Figure 1). It is plausible that deletion of the BIR domain of tLivin would lead to a more efficient induction of apoptosis in A549 and MelA1 cells as well.

JNK is a MAPK protein that plays a role in a signaling cascade that converts extracellular signals into a variety of cellular responses. The documented involvement of the JNK pathway in multiple forms of cell death [28], [31]–[33], [65] coupled with the documented interaction of JNK with Livin [41] prompted us to examine its role in both tLivin-induced apoptosis and necrosis. We show that tLivin induces the activation of JNK in both necrotic cell death of 293T cells and apoptotic cell death of MelA1 cells (Figure 5). Additionally, we demonstrate that both tLivin-induced necrosis and apoptosis are partially dependent on the activation of the JNK pathway (Figure 6) indicating that activation of JNK is required for complete induction of cell death by tLivin. The tLivinΔBIR mutant did not induce activation of JNK in 293T cells (Figure 5), which may point to a role of the BIR domain of tLivin in the activation of JNK.

tLivin has proved to be a strong inducer of cell death in every cell line tested [15], [64] (Figures 1–3). This is possibly due to its ability to induce more than one form of cell death, as we have shown in our work. The apoptotic machinery is frequently damaged in cancer cells; therefore, the ability of tLivin to demonstrate flexibility in induction of cell death and activate an alternative non-apoptotic form of cell death, ensures efficient killing of these cells. This feature of tLivin may be harnessed for the development of new tLivin-based anti-cancer drugs.
